# Aging impacts transcriptomes but not genomes of hormone-dependent breast cancers

**DOI:** 10.1186/bcr1765

**Published:** 2007-09-12

**Authors:** Christina Yau, Vita Fedele, Ritu Roydasgupta, Jane Fridlyand, Alan Hubbard, Joe W Gray, Karen Chew, Shanaz H Dairkee, Dan H Moore, Francesco Schittulli, Stefania Tommasi, Angelo Paradiso, Donna G Albertson, Christopher C Benz

**Affiliations:** 1 Buck Institute for Age Research, 8001 Redwood Boulevard, Novato, CA 94945, USA; 2 University of California Comprehensive Cancer Center, 2340 Sutter Street, University of California, San Francisco, CA 94143, USA; 3 California Pacific Medical Center Research Institute, 475 Brannan Street, San Francisco, CA 94107, USA; 4 National Cancer Institute – Bari, via Amendola 209, 70126 Bari, Italy

## Abstract

**Introduction:**

Age is one of the most important risk factors for human malignancies, including breast cancer; in addition, age at diagnosis has been shown to be an independent indicator of breast cancer prognosis. Except for inherited forms of breast cancer, however, there is little genetic or epigenetic understanding of the biological basis linking aging with sporadic breast cancer incidence and its clinical behavior.

**Methods:**

DNA and RNA samples from matched estrogen receptor (ER)-positive sporadic breast cancers diagnosed in either younger (age ≤ 45 years) or older (age ≥ 70 years) Caucasian women were analyzed by array comparative genomic hybridization and by expression microarrays. Array comparative genomic hybridization data were analyzed using hierarchical clustering and supervised age cohort comparisons. Expression microarray data were analyzed using hierarchical clustering and gene set enrichment analysis; differential gene expression was also determined by conditional permutation, and an age signature was derived using prediction analysis of microarrays.

**Results:**

Hierarchical clustering of genome-wide copy-number changes in 71 ER-positive DNA samples (27 younger women, 44 older women) demonstrated two age-independent genotypes; one with few genomic changes other than 1q gain/16q loss, and another with amplifications and low-level gains/losses. Age cohort comparisons showed no significant differences in total or site-specific genomic breaks and amplicon frequencies. Hierarchical clustering of 5.1 K genes variably expressed in 101 ER-positive RNA samples (53 younger women, 48 older women) identified six transcriptome subtypes with an apparent age bias (*P *< 0.05). Samples with higher expression of a poor outcome-associated proliferation signature were predominantly (65%) younger cases. Supervised analysis identified cancer-associated genes differentially expressed between the cohorts; with younger cases expressing more cell cycle genes and more than threefold higher levels of the growth factor amphiregulin (*AREG*), and with older cases expressing higher levels of four different homeobox (*HOX*) genes in addition to ER (*ESR1*). An age signature validated against two other independent breast cancer datasets proved to have >80% accuracy in discerning younger from older ER-positive breast cancer cases with characteristic differences in *AREG *and *ESR1 *expression.

**Conclusion:**

These findings suggest that epigenetic transcriptome changes, more than genotypic variation, account for age-associated differences in sporadic breast cancer incidence and prognosis.

## Introduction

Age is the strongest demographic risk factor for most human malignancies, including breast cancer [1]. About 80% of all breast cancers occur in women older than age 50; the 10-year probability of developing invasive breast cancer increases from <1.5% at age 40, to about 3% at age 50 and to >4% by age 70, resulting in a cumulative lifetime risk of 13.2% (one in eight) and a near ninefold higher incidence rate in women older than age 50 as compared with their younger counterparts [2,3]. Despite awareness that breast cancer and other cancers are primarily age-related diseases, molecular and cellular hypotheses explaining the cancer–aging relationship have only recently emerged and remain clinically unproven [4].

At the subcellular level, normal human aging has been linked to increased genomic instability [5,6], to global and promoter-specific epigenetic changes [7,8], and to altered expression of genes involved in cell division and extracellular matrix remodeling [5,6]. These associations have led to the hypothesis that the cancer-prone phenotype of an older individual results from the combined effects of cumulative mutational load, increased epigenetic gene silencing, telomere dysfunction and altered stromal milieu [9]. Given the worrisome social, economic and medical consequences of an aging worldwide population, proposed biological mechanisms linking cancer with aging must be established in order to develop effective interventions.

As with normal organs and tissues, tumor biology can also change with aging [10,11]. For sporadic breast cancer in particular, correlations between patient age at diagnosis, tumor biology and clinical prognosis have long been appreciated if not fully understood [12-16]. Younger age at diagnosis (≤ 45 years old) is associated with more aggressive breast cancer biomarkers, including overexpression of ERBB2/HER2 and ERBB1/HER1 growth factor receptors [13], abnormal p53 expression [13,15], estrogen receptor (ER) negativity [12-16], higher nuclear grade and higher Ki-67 proliferation index [12-14,16]. These breast cancer biomarkers are also interdependent, however; in particular, ER expression is inversely correlated with abnormal p53 [15], overexpression of ERBB2 [15], high Ki-67 and nuclear grade, and poor patient prognosis [17]. It therefore remains unclear whether the age-specific biomarker features of breast cancer reflect the pleotropic background effects of aging on the normal mammary gland or age-specific differences in breast tumorigenesis; also, since most age-specific biomarkers strongly associate with the ER status, the effects of aging must be studied in histologically similar breast cancer phenotypes controlled for ER status.

The molecular and cellular effects of aging on both normal and malignant breast tissue are superimposed on a continuum of developmental changes that normally occur between puberty and menopause, heavily influenced by menstrual history and parity. In general, the normal mammary gland ER content (fmol receptor/g tissue) as well as the proportion of ER-expressing (ER-positive) ductal epithelial cells increase with each decade of age, and reach a plateau with menopause at about age 50 [18,19]. In contrast, breast cancer ER expression continues to rise beyond menopause, reaching a near 25-fold differential between normal and malignant mammary gland ER expression in patients by age 70 [18].

Curiously, expression of some ER-inducible gene markers, such as progesterone receptor (PR), pS2, Bcl2 and cathepsin D, does not show any significant relationship with the age at diagnosis [13,18], while other markers show increased expression in breast cancers arising earlier in life [20] – suggesting that the effects of aging may in part be attributed to age-related differences in estrogen-inducible ER pathways. Important in this regard is the age-related change in PR coexpression within ER-positive breast cancers, since PR has long been used as a clinical indicator for a functioning ER pathway in tumors likely to respond to endocrine therapy [21]. Among all ethnic patient groups, ER-positive/PR-negative breast cancers show the greatest age-related increase in incidence after age 40 [22]. Potentially relevant to this ER-positive/PR-negative phenotype is the fact that growth-factor-activated pathways downregulate PR expression [22-25], and that the inverse correlation between overexpression of the ERBB2 growth factor receptor and PR positivity is only seen in breast cancers arising after age 40 [26]. Surprisingly, the natural perimenopausal decline in ovarian-produced estrogen serum levels do not fully account for age-related changes in ER-regulated mammary epithelial pathways, since the marked age-related increase in stromal and epithelial aromatase expression produces postmenopausal mammary gland estrogen levels comparable with those measured in premenopausal women [27].

To better understand the molecular and cellular influences of aging on breast cancer biology and clinical behavior, we performed a detailed study of phenotypically similar breast cancers arising in two disparate patient age groups. The DNA and the RNA were prospectively extracted from cryobanked samples of stage-matched and histology-matched ER-positive breast cancers diagnosed in either younger (age ≤ 45 years) or older (age ≥ 70 years) Caucasian women. These samples were analyzed by array comparative genomic hybridization (CGH) and by high-throughput expression microarrays to look for genetic and epigenetic differences between the age cohorts. Unsupervised hierarchical clustering of the combined data from both cohorts was used to search for age biases in clustered subsets, and this was followed by supervised comparisons between the two cohorts to delineate potential age-related genomic and transcriptome differences. Finally, a predictive analysis of microarrays (PAM) performed on the two age cohorts produced an age-specific expression signature that proved to have >80% predictive accuracy when validated against two other independent breast cancer datasets.

## Materials and methods

### Breast cancer samples extracted for DNA and RNA

Cryobanked breast cancer specimens excised from newly diagnosed Caucasian females were obtained from the University of California San Francisco (UCSF) Comprehensive Cancer Center Breast Oncology Program Tissue Core (*n *= 66) and from the National Cancer Institute – Bari (NCI-Bari) (*n *= 71), following multi-institutional review board approvals. Tumor specimen selection criteria included sporadic incidence (no first-degree relatives with breast cancer), >50% invasive cancer cellularity, a frozen wet weight of at least 100 mg, ER-positivity (>10% nuclear immunohistochemical stain), and patient age at diagnosis either ≤ 45 years (younger cases) or ≥ 70 years (older cases). The 66 UCSF cases were all node-negative and were of predominantly ductal histology (60/66); 54 cases were associated with outcome annotation. The 71 NCI-Bari cases were all of ductal histology with mixed nodal status and without any outcome annotation. The two age cohorts within both tumor sets showed no significant imbalances in the stage, the grade, and the PR status or ERBB2 status. PR-positivity was defined as ≥ 10% nuclear immunohistochemical staining, and ERBB2-positivity was defined as gene amplification.

Cryobanked specimens were pulverized under liquid nitrogen prior to nucleic acid extraction. DNA was purified from frozen tumor powders using the High Pure PCR Preparation Kit (Roche Diagnostics, Indianapolis, IN, USA) and its quality was verified by gel electrophoresis. Total RNA was purified using Trizol reagent as per the manufacturer's protocol (Invitrogen, Carlsbad, CA, USA). Some RNA samples initially stored in formamide were further purified through RNeasy columns according to the manufacturer's protocol (QIAGEN, Valencia, CA, USA). All RNA samples were quality verified on a bioanalyzer (Agilent Technologies, Palo Alto, CA, USA). DNA from all 71 NCI-Bari specimens were used for array-based CGH analysis, but only 35 of these specimens also yielded sufficient RNA for expression microarray analysis. The 35 NCI-Bari RNA samples (RNA sample set 1) were therefore combined with 66 RNA samples prepared from the UCSF specimens (RNA sample set 2) to yield 101 total RNA samples (53 younger women, 48 older women) for expression microarray analysis.

### Array comparative genomic hybridization and data processing

Array CGH and data processing were carried out as described in reference [28]. Test and reference genomic DNAs (500–1,000 ng) were labeled with Cy3 and Cy5, respectively, in a random priming reaction using 25–50 μl reaction volumes. The two labeled DNAs together with Cot-1 DNA (100 μg) were hybridized for 48–72 hours at 37°C onto arrays of 2,464 BAC clones, each printed in triplicate (HumArray1.14 and Hum-Array2.0; UCSF Comprehensive Cancer Center Microarray Core). Data from both array versions were combined only for the BAC clones present on both arrays; duplicate clones were averaged and the final dataset contained 2,240 unique BACs. Images were acquired and the data were processed as previously described [28]. For each tumor, data were plotted in genome order as the mean log_2 _ratio of the replicate spots for each clone normalized to the genome median log_2 _ratio. Array CGH data were deposited in the public Gene Expression Omnibus database (GSE8801).

Array CGH data were analyzed using circular binary segmentation [29] with default parameters to translate intensity measurements into regions of equal copy number, as implemented in the DNAcopy R/Bioconductor package [30]. Missing values were imputed using the maximum value of two flanking segments, producing smoothed values. The gain and loss status for each probe was assigned using the mergeLevel procedure [31]. Tumor profiles were clustered using smoothed imputed data with outliers present; and agglomerative hierarchical clustering was performed using the Euclidean distance as a similarity measure and using the Ward method to minimize the sum of variances to produce compact spherical clusters. A clone-wise comparison of the phenotypic groups was made by *t*-testing smoothed values and controlling for the false discovery rate, with a false-discovery-rate-adjusted *P *value ≤ 0.05 for significance. The Kruskal–Wallis rank sum test was used to analyze phenotypic associations with the following autosomal genomic parameters: the number of break points and chromosomes with break points, the number of amplifications and chromosomes with amplifications, the number of whole chromosome changes and the fraction of genomes altered.

The fractions of genome gained and lost were computed for each tumor, and the frequency of alterations at each clone was computed as the proportion of samples showing an alteration at that locus. The amplification status for a clone was determined by considering the width of the segment to which that clone belonged (0, if an outlier) and a minimum difference between the smoothed value of the clone and the segment means of the neighboring segments. A clone was declared amplified if it belonged to the segment spanning less than 20 Mb and the minimum difference was greater than exp(-*x*3), where *x *is the final smoothed value for the clone. This procedure allowed clones with small log_2_-ratio values to be declared amplified if they were high relative to surrounding clones. To calculate the number of chromosomes with amplifications, a chromosome was said to be amplified if at least one of its clones was amplified. Whole chromosome changes were assigned to chromosomes without identified breakpoints if a chromosomal segment mapped to a gain or a loss level. The number of chromosomal break points was calculated as the number of copy number levels within each chromosome across the genome minus the number of chromosomes. To calculate the number of chromosomes with break points, at least one break point per chromosome was necessary.

### Microarray expression profiles and data processing

The total RNA (3–5 μg per sample) was labeled and analyzed using Affymetrix (Santa Clara, CA, USA) HT-HG_U133A Early Access Arrays with 22.9 K probes representing ~13 K unique UniGenes. Analyses were performed by standard Affymetrix procedures within the Lawrence Berkeley National Laboratory and Life Science Divison's Molecular Profiling Laboratory [32]. Probe set measurements were generated from quantified Affymetrix image files (.CEL files) using the RMA algorithm in Bioconductor R. Array data were deposited in the public Gene Expression Omnibus database (GSE7378 and GSE8193).

Gene expression values were mean centered, with a low variation filter applied to exclude probe sets that did not have at least 10 observations exhibiting a twofold change from the mean. Filtered probes were annotated (GeneTraffic annotation file, March 2006) and those with unknown UniGene symbols were omitted, yielding a final significant probe set of 6,632 annotated probes representing 5,109 unique genes. Unsupervised hierarchical clustering of the mean centered significant probe set was performed using Cluster [33], and was visualized with Java TreeView [34]. Phenotypes (for example, age cohort, PR status and ERBB2 status) of the resulting clusters were compared by chi-square test.

Gene set enrichment analysis (GSEA) was performed using GSEA software (version 2.01) [35,36] to assess tumor phenotypes (for example, age cohort, PR status or ERBB2 status) with respect to specific gene signatures including a mitogen-activated protein kinase (MAPK)-regulated gene set [37], a luminal tumor subtype gene set [38] and a proliferation-associated gene set [39]. The enrichment of all curated gene sets (c2) within the Molecular Signature Database [36], satisfying the gene set size-filtering criteria (maximum = 500, minimum = 10), were also evaluated. Ranking of UniGenes within each phenotype was based on a signal-to-noise metric; an enrichment score for each signature was derived as a function of the likelihood of that gene set being among the most highly ranked genes within the phenotype. For genes represented by more than one probe, the median expression level of all corresponding gene probes was used; the significance of the enrichment score was estimated by 1,000 random permutations of the sample labels in the tumor dataset. For gene sets showing statistically significant enrichment with respect to the age cohort (*P *< 0.05), unsupervised clustering was performed on the entire set of RNA samples using those genes; the outcome association of high gene set expressors versus low gene set expressors was also tested by Kaplan–Meier analysis of the 54 cases with known recurrence events.

A conditional permutation test was performed to identify differentially expressed genes with respect to age and conditional on the RNA sample sources (NCI-Bari and UCSF). For each gene, a linear-fit model of expression level versus age cohort was adjusted for the categorical sample source, and the *P *value was based on the *t *statistic on the slope of age. The null distribution used, however, was not the standard normal but was based on permuting the age cohort distribution within each sample source, which provides an exact test of the conditional independence of age given the sample source. This was expected to control for possible different age effects within each sample source, and to adjust for potential spurious associations based on the different sample sources [40]. Reported associations between the differentially expressed genes and either cancer or aging were determined using the MEDGENE database [41,42], entering the following search terms: Neoplasm, Breast Neoplasms, Carcinomas, Breast Carcinomas, and Aging, Premature. Functional annotation of the differentially expressed genes was performed by gene ontology (GO) analysis [43]; significant enrichment for specific biological functional categories (≥ 5 probes within a process class, Expression Analysis Systematic Explorer [44] score <0.05) was identified from the DAVID database [43,45].

An age cohort gene signature was obtained by training PAM software [46] on RNA sample set 2 (UCSF), minimizing the cross-validation error for the individual age cohorts. The resulting PAM classifier was used to predict the age cohort of RNA sample set 1 (NCI-Bari). For external validation purposes, expression microarray data from Miller and colleagues [47] and from Sotiriou and colleagues [48] were downloaded from the NCBI public Gene Expression Omnibus database (GSE3494 and GSE2990). ER-positive cases with age characteristics matching our age cohorts were selected, resulting in 102 cases from Miller and colleagues' study and 47 cases from Sotiriou and colleagues' study. As the training set (RNA sample set 2) comprised only early-stage tumors, PAM validation was also performed on the external datasets restricted to only ER-positive, node-negative cases (35 cases from Sotiriou and colleagues [48], and 64 cases from Miller and colleagues [47]). Since these microarray studies used Affymetrix U133A platforms, probe set measurements were generated using the RMA algorithm in Bioconductor R and the resulting data were mapped to our significant gene set by Affymetrix probe identifiers, to which the PAM classifier was applied. The significance of the prediction accuracy was determined using Fisher's exact test.

## Results

### Age and ER-positive breast cancer genomic profiles

**Figure 1:**
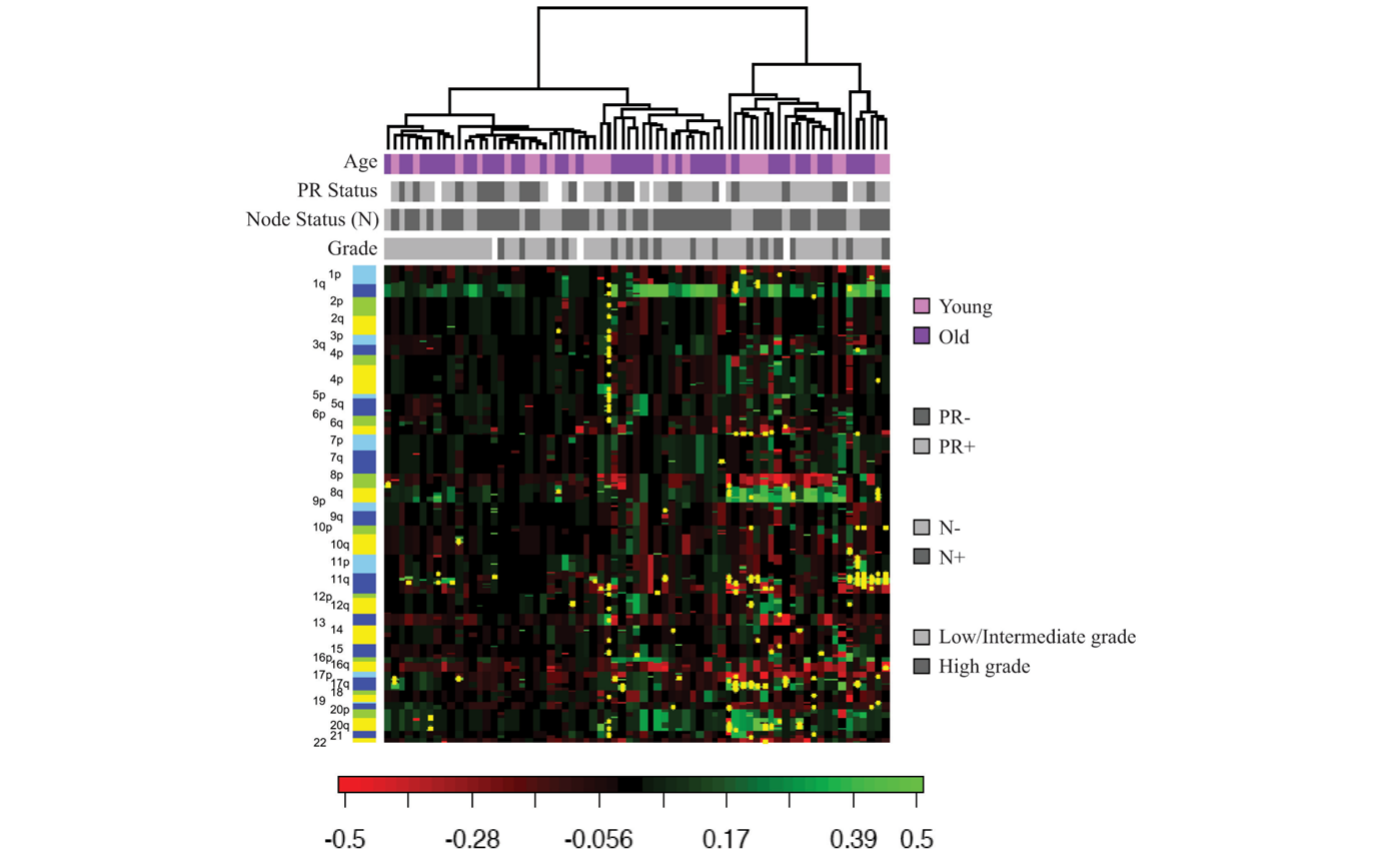
Hierarchical clustering of primary estrogen receptor-positive breast cancers based on genome-wide DNA copy number aberrations. Unsupervised hierarchical clustering of 71 primary estrogen receptor-positive breast cancers, diagnosed in younger women (age ≤ 45 years) or older women (age ≥ 70 years), based on genome-wide DNA copy number aberrations. As previously reported for BAC-based array comparative genomic hybridization analyses of human breast cancer samples [5], columns represent individual tumor samples and rows represent individual genome probes (BAC clones), ordered by chromosome and genome position with 1pter at the top and 22qter at the bottom. Chromosome p-arms and q-arms are shown as different shades of the same color (blue = odd numbered chromosomes, yellow = even numbered chromosomes). As indicated in the color scale at the bottom, genome copy number losses are indicated in red (-0.5) and copy number gains are indicated in green (0.5). Yellow dots represent high-level genomic amplifications. Colored and grey-toned upper bars identify the age cohort, progesterone receptor (PR) status, nodal status and grade status of the estrogen receptor-positive samples in each column. The dendrogram shows unsupervised classification of the 71 samples into two primary clusters and four secondary clusters, with no significant cluster bias according to age, PR status, nodal status or grade status (*P *> 0.3, Fisher exact test).

Array CGH at 1 MB resolution was performed on 71 ER-positive primary breast cancer DNA samples from the two age cohorts: 27 younger cases and 44 older cases, matched for stage and histology (nodal status, grade, PR status). Unsupervised hierarchical clustering of the genome-wide copy number changes (smoothed log_2 _ratios), as shown in Figure 1, revealed that these ER-positive cancers comprise two basic genome aberration patterns that have been previously characterized from unselected breast cancer collections [28]: a simple genotype with few genomic copy number changes other than gain of 1q and loss of 16q, and a mixed amplifier genotype with recurrent amplifications and low-level genomic gains and losses. Neither the two primary dendrogram clusters representing these two basic genotypes nor any of the secondary dendrogram clusters exhibit any bias (*P *> 0.3, Fisher exact test) with respect to age, nodal status or PR status. Direct comparison of the two age cohorts for multiple array CGH parameters revealed no significant differences in the number of break points, chromosomes with break points, amplifications, chromosomes with amplifications, or whole chromosome changes; also, the fraction of genome gained, lost or otherwise altered were not significantly different between the two age cohorts (Additional file 1). While nonsignificant trends suggested slightly fewer oncogene amplifications within the older cohort, overall amplification frequencies for the most common oncogene loci were as follows: *MYC *(8q24.2; 27%), *CCND1 *(11q13.3; 23%), *ZNF217 *(20q32; 17%), *AIB1 *(20q13.12; 16%), *MDM2 *(12q15; 8%), *ESR1 *(6q25; 7%), *ERBB2 *(17q12; 7%), and *TOPO2A *(17q21; 7%).

### Unsupervised analysis of ER-positive breast cancer expression profiles

**Figure 2:**
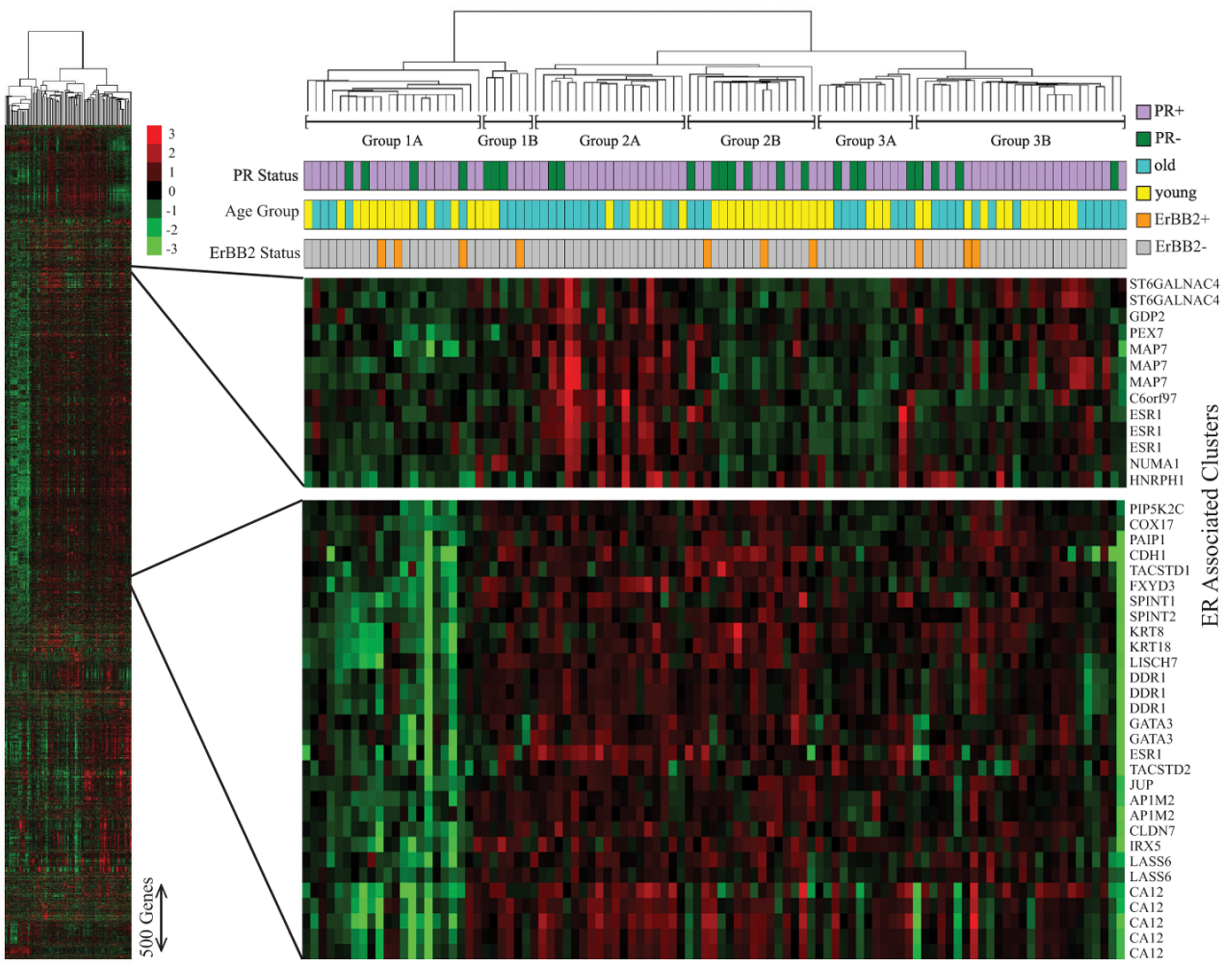
Hierarchical clustering of primary estrogen receptor-positive breast cancers based on genome-wide microarray profiling. Unsupervised hierarchical clustering of 101 primary estrogen receptor (ER)-positive breast cancers, diagnosed in younger women (age ≤ 45 years) or older women (age ≥ 70 years), based on genome-wide microarray profiling of 6,632 variably expressed probes (~5.1 K unique genes). The cluster dendrogram defines six different transcriptome subtypes of ER-positive breast cancers (Group 1A, 1B, 2A, 2B, 3A, 3B), with significant age biases (*P *< 0.05) but not biased by progesterone receptor (PR) status or ERBB2 status; horizontal colored bars identify the age cohort, PR status and ERBB2 status of the ER-positive samples in each column. The vertical red–green color scale shows log_2 _ratios from mean centered gene expression levels. Magnified views show ESR1-containing (ER-associated) probe sets within the entire cluster diagram.

RNA sample set 1 (35 NCI-Bari RNA samples) and RNA sample set 2 (66 age-matched UCSF samples) were combined to yield 101 RNA samples from ER-positive breast cancers, arising in the predefined younger (*n *= 53) and older (*n *= 48) age groups, well balanced for tumor size, nodal involvement, grade, PR status and ERBB2 status. Figure 2 shows the unsupervised hierarchical clustering of these 101 breast cancer cases, based on their gene expression similarity across nearly 5.1 K variably expressed unique genes (6,632 probe sets), into six different transcriptome clusters of ER-positive breast cancer (groups 1A, 1B, 2A, 2B, 3A and 3B). The four different *ESR1 *probes on the array defined two primary *ESR1*-associated probe set clusters, including genes (for example, *GATA3*, *KRT8, KRT18*) commonly used to define luminal-type breast cancers [38,39,49-51]. There was an average 65-fold range in the *ESR1 *transcript levels across this entire collection of 101 breast cancers, with the *ESR1*-associated probes showing similar variations. The six different transcriptome clusters showed no significant bias with respect to tumor PR status and ERBB2 status; in contrast, two of the clusters were composed primarily of younger cases (64% of group 1A, 81% of group 2B) and one was composed primarily of older cases (68% of group 2A).

When the cluster compositions with respect to age, PR status and ERBB2 status were statistically compared, only the age cohort distribution was found to be significantly different (*P *< 0.05, chi-square test). Patient outcome data were available on 54 (30 younger, 24 older) of the 101 cases, scattered evenly among the six transcriptome clusters. Kaplan–Meier probability curves for recurrence-free survival indicated that younger age and PR-negative status were associated with earlier relapse, but these outcome differences did not quite reach statistical significance (age, *P *= 0.09; PR status, *P *= 0.08; log-rank analyses). Kaplan–Meier curves for cases representing each of the six transcriptome clusters, however, did achieve significant separation (*P *= 0.025, log-rank analysis), with the predominantly younger group 2B cases showing the shortest survival (median recurrence-free survival = 2.5 years) and the predominantly older group 2A cases showing significantly more prolonged survival (median recurrence-free survival = 6.2 years).

### Gene set enrichment analysis

Probe sets for the variably expressed genes were subjected to GSEA with respect to each age cohort for MAPK-regulated genes [37], luminal subtype markers [38] and a gene proliferation signature [39] (gene signatures in Additional file 2). As shown in Table 1, in addition to comparing the age cohorts, these gene signatures were used to compare PR-negative cases versus PR-positive cases and to compare ERBB2-negative cases versus ERBB2-positive cases. There was no enrichment of any of the three gene signatures according to the tumor PR status; and only the proliferation signature showed any significant relationship to the ERBB2 status, with these proliferation genes more highly expressed in the ERBB2-positive breast cancer cases (nominal *P *= 0.01; adjusted for multiple comparisons *P *= 0.02). Neither MAPK-upregulated nor MAPK-downregulated genes showed any significant relationship with age cohort, PR status or ERBB2 status when multiple gene set testing was taken into account. Luminal markers, commonly used as an expression array signature for ER-positive breast cancers, showed no significant relationship with age cohort, ERBB2 status or PR status, although there was a nonsignificant trend for luminal gene expression to associate with PR-positive cases. Proliferation genes were significantly more highly expressed in the younger cohort (nominal *P *= 0.006; adjusted for multiple comparisons *P *= 0.011). Interestingly, of the other 1,176 c2:curated gene sets (passing through the size filter) similarly evaluated, none showed significant enrichment according to age, tumor PR status or ERBB2 status when multiple testing was accounted for, although two notable trends were observed: cell cycle genes, as annotated by GO (c2:500), were enriched in the younger age cohort (nominal *P *= 0.006, family-wise error rate *P *= 0.118); and early response genes downregulated by enforced expression of a naturally transforming chimeric HOX developmental gene, NUP98-HOXA9 (c2:934) [52], were enriched in the older age cohort (nominal *P *= 0.000, family-wise error rate *P *= 0.112).

**Table 1:** Gene set enrichment analysis results for the gene list tested

Gene set	Comparison								
	
	Old over young cohort			Progesterone receptor-negative over progesterone receptor-positive tumors			ERBB2-negative over ERBB2-positive tumors		
	
	Enrichment score	Nominal *P *value	FWER *P *value	Enrichment score	Nominal *P *value	FWER *P *value	Enrichment score	Nominal *P *value	FWER *P *value
MAPK upregulated	0.44	0.05	0.13	0.28	0.49	0.67	0.28	0.46	0.65
MAPK downregulated	-0.36	0.17	0.4	-0.31	0.33	0.57	0.26	0.26	0.68
Luminal markers	0.31	0.72	0.79	-0.63	0.1	0.15	0.48	0.38	0.57
Proliferation markers	-0.85	0.006	0.011	0.62	0.32	0.38	-0.83	0.01	0.02

Gene sets showing significant enrichment after adjustment for multiple gene set testing (family-wise error rate (FWER) *P *< 0.05) are presented in bold.

**Figure 3:**
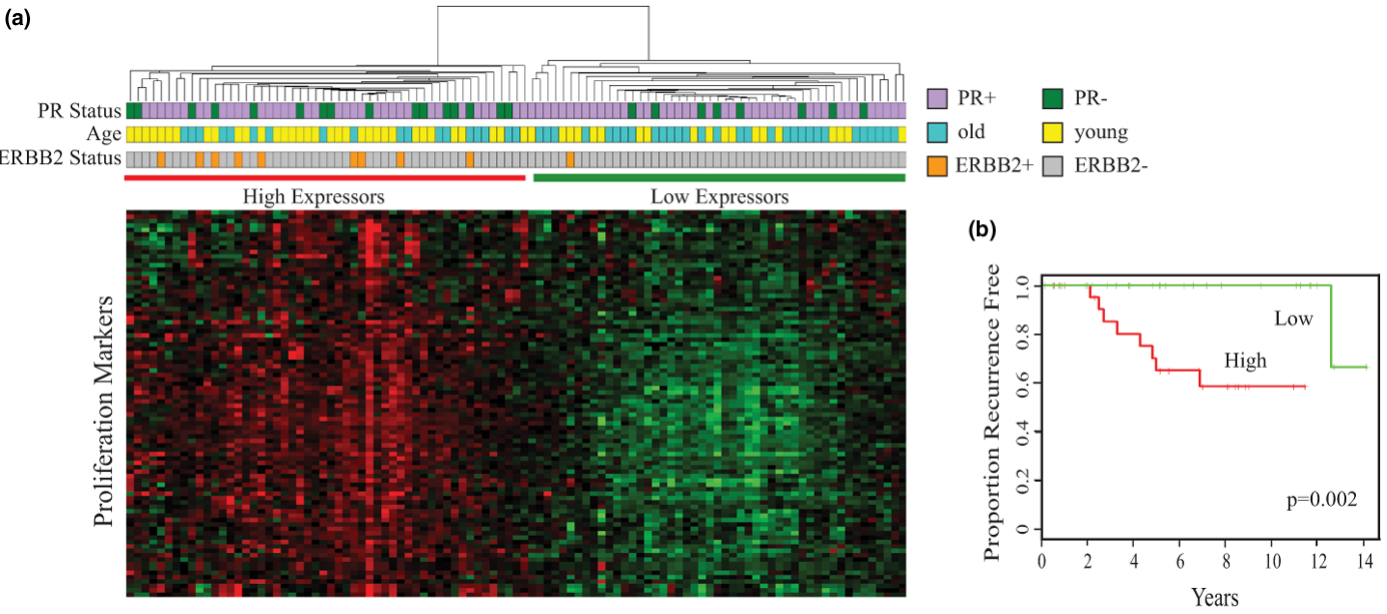
Estrogen receptor-positive breast cancer subsets by gene set enrichment analysis. Assessment of estrogen receptor (ER)-positive breast cancer subsets by gene set enrichment analysis (GSEA) for specific gene signatures. **(a) **Unsupervised clustering of the 101 primary ER-positive breast cancers shown in Figure 2 based only on expression of the 71-gene proliferation signature shown to be significant by GSEA, revealing two major clusters (high expressors and low expressors of proliferation signature) with significant biases in age and ERBB2 status; horizontal colored bars identify the age cohort, progesterone receptor (PR) status and ERBB2 status of the samples in each column. **(b) **Kaplan–Meier plots of recurrence events among the 54 ER-positive cases with known clinical follow-up, dichotomized by high (red) or low (green) expression of the 71-gene proliferation signature, with significance determined by log-rank analysis.

As shown in Figure 3a, when the proliferation genes were used to perform unsupervised hierarchical clustering of the 101 cases, two comparably sized subsets were identified. The subset with more highly expressed proliferation genes contained most of the younger age cases (34/52) and all but one of the ERBB2-positive cases (*P *< 0.05, chi-square test). These proliferation genes were also used to dichotomize the 54 cases with known clinical outcome; as shown in the Kaplan–Meier curves in Figure 3b, cases with more highly expressed proliferation genes exhibited significantly worse recurrence-free survival (*P *= 0.002, log-rank analysis).

### Differential gene expression between age cohorts

A conditional permutation strategy was used to identify 75 unique genes (84 probe sets) differentially expressed between younger and older cohorts (false discovery rate *P *< 0.05), presented in Table 2. Of these genes, 24 genes (28 probes) showed increased expression in the younger cohort relative to the older cohort (including *GREB1 *and *AREG*), while 51 genes (56 probes) showed increased expression in the older cohort relative to the younger cohort (including *ESR1*). Interestingly, the estrogen-responsive genes, *GREB1 *and *AREG*, showed higher expression in the younger cohort, which showed significantly lower *ESR1 *expression.

**Table 2:** Differentially expressed genes between the young and old cohorts

UniGene symbol	Fold change^a^	UniGene name
Genes with higher expression in the young cohort		
AREG^b^	3.12	Amphiregulin (schwannoma-derived growth factor)
PRSS2^b^	2.71	Protease, serine, 2 (trypsin 2)
GREB1^b^	2.34	GREB1 protein
PTHLH^b,d^	2.01	Parathyroid hormone-like hormone
HPGD^c^	1.98	Hydroxyprostaglandin dehydrogenase 15-(NAD)
SPANXA1///SPANXB1///SPANXA2///SPANXC^c^///SPANXB2	1.91	Sperm protein associated with the nucleus, X-linked, family member A1///SPANX family, member B1///SPANX family, member A2///SPANX family, member C///SPANX family, member B2
LAMA3^d^	1.87	Laminin, alpha 3
ATP6V1B1^c^	1.82	ATPase, H^+ ^transporting, lysosomal 56/58 kDa, V1 subunit B, isoform 1 (renal tubular acidosis with deafness)
S100A2^b,d^	1.79	S100 calcium binding protein A_2_
DIO2^c^	1.79	Deiodinase, iodothyronine, type II
PRSS1^b^///PRSS2^b^///PRSS3///TRY6	1.77	Protease, serine, 1 (trypsin 1)///protease, serine, 2 (trypsin 2)///protease, serine, 3 (mesotrypsin)///trypsinogen C
FGFR1^b,d,e^	1.65	Fibroblast growth factor receptor 1
TP73L^b,d^	1.60	Tumor protein p73-like
PRSS1^b^	1.59	Protease, serine, 1 (trypsin 1)
C20orf59	1.57	Chromosome 20 open reading frame 59
DLG7^b,f^	1.56	Discs, large homolog 7 (Drosophila)
ELOVL2^c^	1.52	Elongation of very long chain fatty acids (FEN1/Elo2, SUR4/Elo3, yeast)-like 2
KIF2C^b,f^	1.50	Kinesin family member 2C
STK6^b,f^	1.49	Serine/threonine kinase 6
UST^c^	1.47	Uronyl-2-sulfotransferase
CDC14A^f^	1.42	CDC14 cell division cycle 14 homolog A (*S. cerevisiae*)
ELL3^c^	1.42	Elongation factor RNA polymerase II-like 3
RAD54B^c,f^	1.41	RAD54 homolog B (*S. cerevisiae*)
ITGA2^b,d,e^	1.33	Integrin, alpha 2 (CD49B, α_2 _subunit of VLA-2 receptor)
Genes with higher expression in the old cohort		
HOXB6^b,d^	2.40	Homeobox B6
TMC5	2.36	Transmembrane channel-like 5
HOXB2^c,d^	1.99	Homeobox B2
ST6GALNAC5	1.99	ST6 (α-*N*-acetyl-neuraminyl-2,3-β-galactosyl-1,3)-*N*-acetylgalactosaminide α-2,6-sialyltransferase 5///ST6 (α-*N*-acetyl-neuraminyl-2,3-β-galactosyl-1,3)-*N*-acetylgalactosaminide α-2,6-sialyltransferase 5
KIAA1102	1.82	KIAA1102 protein
PYGL^c^	1.82	Phosphorylase, glycogen; liver (Hers disease, glycogen storage disease type VI)
TNFSF10^b^	1.82	Tumor necrosis factor (ligand) superfamily, member 10///tumor necrosis factor (ligand) superfamily, member 10
GOLPH2^c^	1.78	Golgi phosphoprotein 2
DSPG3^c^	1.75	Dermatan sulfate proteoglycan 3
GLRX^c^	1.75	Glutaredoxin (thioltransferase)
FLJ20152	1.73	Hypothetical protein FLJ20152
GATM^c^	1.72	Glycine amidinotransferase (l-arginine:glycine amidinotransferase)
ENTPD5^b^	1.69	Ectonucleoside triphosphate diphosphohydrolase 5
SASH1^b,f^	1.69	SAM and SH3 domain containing 1
ITPR1^b^	1.68	Inositol 1,4,5-triphosphate receptor, type 1
ANG^c,d,e^///RNASE4	1.66	Angiogenin, ribonuclease, RNase A family, 5///ribonuclease, RNase A family, 4
IQGAP2^b^	1.63	IQ motif containing GTPase activating protein 2
MANSC1	1.62	MANSC domain containing 1
HOXB5^c,d,e^	1.60	Homeobox B5
FAH^b^	1.60	Fumarylacetoacetate hydrolase (fumarylacetoacetase)
ARHGDIB^c,d^	1.60	Rho GDP dissociation inhibitor (GDI) beta
TAPBPL^c^	1.59	TAP binding protein-like
CLMN^c^	1.56	Calmin (calponin-like, transmembrane)
ESR1^b,d,e,f^	1.56	Estrogen receptor 1
EFNA1^b^	1.56	Ephrin-A1
COBLL1	1.56	COBL-like 1
P8^b,d,e^	1.55	p8 protein (candidate of metastasis 1)
SC5DL	1.52	Sterol-C5-desaturase (ERG3 δ-5-desaturase homolog, fungal)-like
CLEC5A	1.52	C-type lectin domain family 5, member A
SEPT6^f^	1.52	Septin 6
RHOB^b,d,e,f^	1.52	Ras homolog gene family, member B
CYB5	1.51	Cytochrome b-5
PDE4A^b^	1.50	Phosphodiesterase 4A, cAMP-specific (phosphodiesterase E2 dunce homolog, Drosophila)
C21orf25	1.49	Chromosome 21 open reading frame 25
CCL3^c^///CCL3L1///CCL3L3	1.49	Chemokine (C–C motif) ligand 3///chemokine (C–C motif) ligand 3-like 1///chemokine (C–C motif) ligand 3-like 3
CCDC28A	1.46	Coiled-coil domain containing 28A
CALM3	1.46	Calmodulin 3 (phosphorylase kinase, delta)
PPFIBP2^c^	1.46	PTPRF interacting protein, binding protein 2 (liprin β2)
DBI^c^	1.46	Diazepam binding inhibitor (GABA receptor modulator, acyl-Coenzyme A binding protein)
SLC25A12^c^	1.45	Solute carrier family 25 (mitochondrial carrier, Aralar), member 12
CPM^b,d,e^	1.44	Carboxypeptidase M
MARCH8	1.43	Membrane-associated ring finger (C3HC4) 8
FLJ20298	1.41	FLJ20298 protein
SLC12A8^c^	1.40	Solute carrier family 12 (potassium/chloride transporters), member 8
FUCA1^b^	1.39	Fucosidase, alpha-L-1, tissue
LOC57146	1.38	Promethin
RANBP2^c^	1.36	RAN binding protein 2
HOXB7^b,d^	1.36	Homeobox B7
PANX1^c^	1.33	Pannexin 1
TGOLN2^b^	1.31	*trans*-Golgi network protein 2
VWA1^c^	1.29	von Willebrand factor A domain containing 1

^a^Young/old for genes with higher expression in the young cohort; old/young for genes with higher expression in the old cohort. ^b^Genes showing first-degree association with cancer and ^c^genes showing second-degree association with cancer (associations as determined through MEDGENE database searches with terms Carcinoma, Ductal Breast Carcinoma, Neoplasms and Breast Neoplasms). ^d^Genes involved in 'development', ^e^genes involved in morphogenesis and ^f^genes involved in the cell cycle, as functionally annotated by the DAVID database and contributed to the enrichment probability calculations – these three major gene ontology biological processes were found to be enriched among the 75 differentially expressed genes. Enrichment was defined as Expression Analysis Systematic Explorer score < 0.05.

A MEDGENE database search (disease terms 'neoplasms', 'breast neoplasms', 'carcinomas', or 'carcinoma, ductal, breast') indicated that 29 of the 75 differentially expressed genes had a published first-degree association with cancer; in contrast, none of the 75 genes had any published association with aging (disease terms 'aging, premature'). GO analysis was used to determine whether the 75 genes differentially expressed between the age cohorts were enriched for specific biological processes. Several functional categories were significantly over-represented (Expression Analysis Systematic Explorer score < 0.05) in this gene set [43], including development, cell cycle, M-phase, morphogenesis and reproduction. As noted in Table 2, 17 of the genes were associated with development and nine genes were associated with the cell cycle, including five genes specifically associated with the M-phase.

### Defining and validating an age cohort signature in ER-positive breast cancers

**Figure 4:**
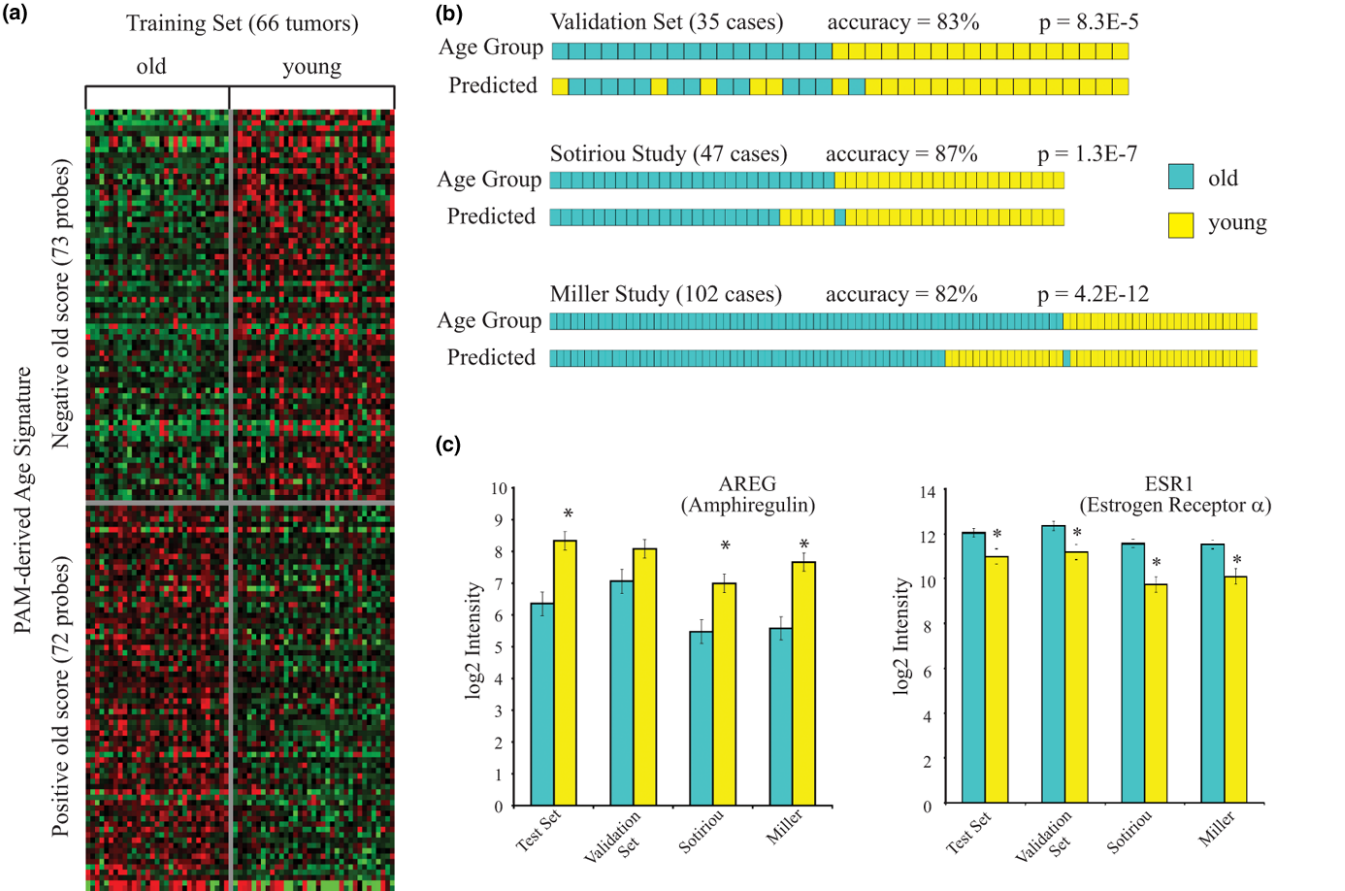
Prediction analysis of microarrays-derived age signature validated against independent estrogen receptor-positive breast cancer datasets. **(b) **University of California San Francisco (UCSF) RNA sample set 2 (*n *= 66, younger women and older women) was used to train prediction analysis of microarrays (PAM) and to derive a 145-probe (128-gene) age cohort classifying signature, arranged in ascending order of the PAM score for cases in the older cohort. **(b) **Actual and signature-predicted age cohort designations for the validating UCSF RNA sample set 1 (*n *= 35) and two external validating datasets restricted to estrogen receptor-positive cases with identical age cohort characteristics: Sotiriou and colleagues [48] and Miller and colleagues [47]. Prediction accuracies are indicated, with Fisher's exact test *P *values presented for significance. **(c) **Age-signature-defined subsets from all four sample datasets show similar differences in log_2 _expression levels (mean ± standard deviation) of *AREG *and *ESR1*.

PAM was applied to RNA sample set 2 to derive an age cohort signature consisting of 128 unique genes (145 probes), one-half of which were overexpressed in the younger cohort relative to the older cohort and one-half of which were overexpressed in the older cohort relative to the younger cohort (Figure 4a). This signature (Additional file 3) was first validated against RNA sample set 1 and was then independently validated against two external breast cancer microarray datasets that included 102 ER-positive cases from Miller and colleagues [47] and 47 ER-positive cases from Sotiriou and colleagues [48] fitting our age selection criteria. The PAM-derived age signature correctly identified older from younger ER-positive cases in all three validation sample sets with comparable accuracy >80% (Figure 4b), and with high statistical certainty (*P *= 8.3 × 10^-5 ^to 4.2 × 10^-12^). Interestingly, the majority of errors were misclassifications of cases from the older cohorts as younger cases. Misclassification bias could not be associated with either nodal status or outcome differences in the Miller and colleagues and Sotiriou and colleagues datasets; in fact, when these external validation datasets were further restricted to node-negative cases to match the training set, only a modest increase was observed in predictive accuracy at the expense of statistical certainty (90%, *P *= 1.7 × 10^-4 ^for Sotiriou and colleagues [48]; 86%, *P *= 1.6 × 10^-7 ^for Miller and colleagues [47]).

*ESR1 *and *AREG *were among the 44 genes in common between the age signature gene set and the differentially expressed gene set; and, as shown in Figure 4c, age-signature-defined subsets from all four sample datasets showed similar differences in mean expression levels (log_2 _intensity) for both *ESR1 *and *AREG*. Of note, a PR signature was similarly derived from RNA sample set 2 (103 probes; Additional file 4), but showed only 67% accuracy (*P *= 0.1) in predicting the PR status of RNA sample set 1.

## Discussion

Although there have been numerous studies of clinical factors addressing the relationship between age at diagnosis and breast cancer prognosis [12,14,16,53-55], few studies have comprehensively investigated the age dependency of the many well-established prognostic breast cancer biomarkers, and no studies have used a prospective study design [13,18]. Concerned about the established inverse relationship between the ER status and poor-risk biomarker surrogates of breast cancer proliferation and genomic instability [13,18], the present study aimed to identify genomic and transcriptome changes associated with aging using DNA and RNA prospectively collected from stage-matched and histology-matched ER-positive breast cancers from younger women (age ≤ 45 years) and older women (age ≥ 70 years), analyzed by array CGH and high-throughput expression microarrays.

Similar bioinformatics-based approaches have been used to characterize aging effects in human fibroblasts [5,6], lymphocytes [5] and myoblasts [56]; however, comparable efforts to investigate aging influences on human cancer biology have not been reported. Moreover, while ER-positive breast cancers have been well studied as a subgroup within unselected breast cancer phenotypes using array CGH [28,57] or expression profiling [38,39,49-51], the present study represents the largest study reported to date using these powerful techniques to subset ER-positive breast cancers, while employing a statistical design powered to detect age-specific differences.

Array CGH analysis of 71 DNA samples confirmed that our ER-positive breast cancers were composed of two basic genotypes [28]: a simple subtype characterized by few genomic copy number changes other than gain of 1q and loss of 16q, and a mixed amplifier subtype characterized by recurrent amplifications but otherwise low levels of genomic gains and losses. A third genomic subtype of breast cancer, referred to as complex, known to be almost exclusively composed of ER-negative breast cancers [28], was not observed in either of the two age cohorts studied. Neither the simple nor the mixed amplifier genomic subtypes of ER-positive breast cancer showed any particular age bias. Direct comparison of the two age cohorts for multiple array CGH parameters also revealed no significant differences in the fraction of genome altered, in whole chromosome changes or in total or site-specific amplicon frequencies. Although nonsignificant trends suggested slightly fewer oncogene amplifications within the older cohort, overall amplification frequencies for the most common oncogenes were as expected for ER-positive breast cancers [51,58]: *MYC *(27%), *CCND1 *(23%), *ZNF217 *(17%), *AIB1 *(16%), *MDM2 *(8%), *ESR1 *(7%), *ERBB2 *(7%), and *TOPO2A *(7%). At the level of genomic resolution (~1 MB) achievable by BAC-based array CGH, there appeared to be few if any genetic differences between ER-positive breast cancers arising in women whose ages differ by more than 25 years. Future studies employing higher density genomic arrays are warranted to confirm this conclusion.

Microarray profiling of 101 RNA samples showed an average 65-fold range in *ESR1 *transcript levels across the entire collection of ER-positive breast cancers, with the older cohort showing significantly higher *ESR1 *levels as compared with the younger cohort, consistent with earlier biomarker studies [13]. There was the expected close correlation between the *ESR1 *transcript levels and commonly observed *ESR1 *coexpressed genes (for example, *GATA3*) as well as other genes (for example, *KRT8*, *KRT18*) that characteristically define luminal-type breast cancer, although this tumor collection also contained several ERBB2-positive cases (10/101) that are not characteristically found in microarray-defined clusters of luminal-type breast cancer [38,39,49-51]. Hierarchical clustering of the ~5.1 K variably expressed genes also identified six transcriptome subtypes of ER-positive breast cancer with significant age biases (*P *< 0.05) but not associated with differing PR status. Based on relapse-free survival analyses of the 54 cases with known clinical outcome (30 younger women, 24 older women), there was a trend supporting a less favorable prognosis for the younger age cases (*P *= 0.09) and PR-negative cases (*P *= 0.08). The six age-biased transcriptome clusters, however, showed significantly different relapse-free survival outcomes (*P *= 0.025, log-rank analysis), suggesting that these transcriptome subtypes represent clinically relevant phenotypes of ER-positive breast cancer. Previous expression array studies analyzing fewer ER-positive cases have identified no more than two or three subsets of luminal-type breast cancer [38,39,49-51].

Reported gene signatures representing luminal, proliferation and MAPK markers were tested for their enrichment in one or the other of the age-stratified cohorts, and only the proliferation gene signature showed any significant age bias when multiple testing was accounted for, being more highly expressed in the younger cohort. This finding is consistent with earlier studies showing higher tumor grade and proliferation markers (for example, mitotic index and Ki-67 positivity) in younger age breast cancer patients [13]. While none of the >1,000 curated gene sets in the Molecular Signature Database that were similarly evaluated demonstrated any significant age biases when multiple testing was account for, a trend was observed for enrichment of cell cycle genes in the younger cohort cases. Nine genes common to both the GO biological process cell cycle set and the proliferation signature set (*BUB1*, *CCNB1*, *CCNE2*, *CDC25A*, *CDC7*, *MAD2L1*, *MCM4*, *ORC6L*, *PTTG1*) were also present in our significant probe set. Among these, four genes (*BUB1*, *CCNE2*, *MAD2L1*, *ORC6L*) have been previously associated with poor-prognosis ER-positive breast cancers in a well-established 70-gene prognostic signature [58]; these genes are therefore probably important contributors to the more aggressive tumor characteristics of ER-positive breast cancers arising in younger patients.

Using only the proliferation gene signature to perform unsupervised hierarchical clustering of the 101 cases generated two comparably sized ER-positive subsets, one with higher expression and another with lower expression of the proliferation genes; the higher expressing subset contained most of the younger age cases (34/52) and all but one of the ERBB2-positive cases. When this proliferation signature was also used to dichotomize the 54 cases with known clinical outcome, the higher expressing cases showed significantly worse disease-free survival as compared with the lower expressing cases, consistent with reports on the association of a similar proliferation signature with poor outcome in patients with ER-positive breast cancer [59]. Interestingly, despite a presumed mechanistic link between activation of growth factor receptors, MAPK signaling and cell proliferation, there was minimal overlap between genes in the reported MAPK and proliferation signatures, and no significant association was observed between the MAPK signature, age and ERBB2 positivity.

Despite the observed positive association between the *ESR1 *expression level and older age, no age association was seen for the luminal gene signature that included *ESR1*, *ESR1*-associated genes and estrogen-inducible genes. This finding is consistent with our previous report showing increased breast cancer ER protein with aging without comparably increased levels of such estrogen-inducible markers as PR, pS2, Bcl2 and cathepsin D [13], and suggesting reduced estrogen signaling in breast tumors of older patients. In keeping with these protein biomarker observations, differential gene expression analysis in the present study did not identify any known estrogen-inducible genes such as *TFF1*, *PGR*, *IRS1*, *IGFBP4*, *PCNA*, *MYC*, *CCNA2 *or *DLEU2 *as being more highly expressed in the older cohort despite higher expression of *ESR1 *in this cohort. In contrast, two estrogen-inducible growth-regulating genes, *GREB1 *and *AREG*, showed significantly higher expression levels in the younger cohort, in keeping with a recent study demonstrating a negative correlation between these estrogen-inducible genes and age [20]. As *GREB1 *and *AREG *are known to induce cell proliferation upon estrogen activation [60,61], their increased expression in the younger cohort offers some mechanistic basis for increased proliferative activity and gene expression in the younger cohort.

Of the 75 unique genes differentially expressed between younger and older cohorts, 24 genes showed increased expression in younger cases relative to older cases (including *GREB1 *and *AREG*) while 51 genes showed increased expression in older cases relative to younger cases (including *ESR1*). Comparison with a well-studied estrogen-inducible gene signature set [20] revealed that ~25% (19/75) of these differentially expressed genes overlapped with known early or late estrogen-responsive genes, and thus potentially reflected hormonal changes associated with menopause rather than aging effects. While two-thirds (13/19) of these potential estrogen-responsive genes showed appropriate directional changes according to cohort menopausal status, supporting this possibility, at least 75% of the differentially expressed genes would appear to be independent of menopausal differences in circulating estrogen levels and, therefore, potentially informative of age-related differences in ER-positive breast cancer biology. A comprehensive database search confirmed that at least 40% of these differentially expressed genes have reported direct links with malignancy; and while none have reported links with premature aging, one of the differentially expressed genes (*KIF2C*) has been previously implicated in aging studies of lymphocytes and fibroblasts [5], while six other genes (*COBLL1*, *HPGD*, *HOXB2*, *PDE4A*, *SLC25A12*, *TP73L*) were recently reported as differentially expressed with age in human skeletal muscle [62].

A search for annotated enrichment of the differentially expressed genes for specific biological processes (GO Biological Processes, Expression Analysis Systematic Explorer score < 0.05) indicated that 'development' and 'cell cycle/M-phase' were the most overrepresented functional gene categories. In keeping with the GSEA observation indicating a trend for enrichment of cell-cycle-associated genes in the younger cohort cases, differentially expressed cell cycle/M-phase genes (including positive regulators such as *STK6*, *FGFR1 *and *DLG7*) represented 20% (5/25) of all genes overexpressed in the younger cohort but only 8% (4/51) of those overexpressed in the older cohort. In contrast, the older cohort cases showed differentially increased expression of negative cell cycle regulators (such as *SASHI *and *RHOB*) and four developmentally essential homeobox genes (*HOXB2*, *HOXB5*, *HOXB6*, *HOXB7*), the latter finding also in keeping with the GSEA observed trend showing enrichment in the older cohort of HOX-regulated (NUP90-HOXA9 repressed) genes. Two of the overexpressed HOXB genes (*HOXB6*, *HOXB7*) have been specifically linked to mammary gland development and are known to be expressed in ER-positive breast cancer cells [63]. *HOXB7*, in particular, known to be dependent on stromal (extracellular matrix) signaling, is transcriptionally upregulated in breast cancers metastatic to bone (relative to primary tumors), and is thought to play a role in promoting angiogenesis, growth factor-independent proliferation and DNA double-strand break repair, conferring breast cancer resistance to the genome destabilizing effects of DNA damage [64].

PAM was used to derive an age signature that consisted of 128 unique genes, including 44 of the 75 differentially expressed genes determined by our conditional permutation approach. The age signature was independently validated against two other age-matched ER-positive breast cancer microarray datasets and proved to have >80% accuracy in distinguishing younger from older ER-positive breast cancer cases. *ESR1 *and *AREG *were among the genes in common between the age signature and the differentially expressed gene sets; it is therefore not surprising that the age-signature-defined subsets from the two independent databases showed similar differences in the mean expression levels of these two genes as found in our age-defined cohorts. Only 28% of the age signature genes overlap with known early or late estrogen-responsive genes, suggesting that this age signature largely reflects age-related differences in the phenotype of ER-positive breast cancer rather than differences in circulating estrogen levels associated with menopausal status.

The fact that a PAM-derived PR signature did not perform well upon validation implies substantial heterogeneity between ER-positive breast cancers with the same PR status, and possibly indicates that confounding age-related gene expression changes are of greater biological importance than PR-related gene expression differences. Misclassification errors using the age signature were more prevalent among the older cohort cases, also suggesting greater variation in expression of the age signature genes with aging. Of further interest, the 128 age signature gene set was unable to accurately subset ER-negative cases identified from the two independent breast cancer datasets [47,48], consistent with expression-array-based conclusions that the biology of ER-positive and ER-negative breast cancers are fundamentally distinct, and supporting the likelihood that the PAM-derived age signature incorporates biological profiles specific to ER-positive breast cancers but not ER-negative breast cancers.

## Conclusion

This prospectively designed study addresses a pressing need to evaluate molecular and cellular hypotheses proposed to explain age-related differences in breast cancer incidence and clinical behavior. It is hard to reconcile the evidence gathered in this study of ER-positive breast cancers with the more general cancer-aging postulate that the breast-cancer-prone phenotype of an older woman results from genomic instability and age-accumulated mutational loads secondary to telomeric dysfunction and/or progressive DNA damage [9]. More consistent with the present evidence is the likelihood that ER-positive breast cancers arising in older women relative to younger women do so by a fundamentally different tumorigenic process, manifested more by epigenetic transcriptome differences such as those regulated by HOX genes, and less by genomic differences that were not detected using state-of-the-art BAC-based CGH analyses. More pronounced expression of cell cycle and proliferation-associated genes emerged as a strong defining feature of ER-positive breast cancers arising in younger women, perhaps even driving their earlier clinical appearance; this observation is certainly consistent with the more aggressive clinical nature of early-age-onset breast cancer.

Age cohort study designs of this type are needed to not only confirm the specific transcriptome differences noted here, but also to look for common age-associated differences in gene classes and functional pathways that may enable us to generalize about the age-related biological differences driving ER-negative breast tumorigenesis as well as the many other age-associated epithelial malignancies other than breast cancer.

## Abbreviations

CGH = comparative genomic hybridization; ER = estrogen receptor; GO = gene ontology; GSEA = gene set enrichment analysis; MAPK = mitogen-activated protein kinase; NCI-Bari = National Cancer Institute – Bari; PAM = prediction analysis of microarrays; PR = progesterone receptor; UCSF = University of California San Francisco.

## Competing interests

The authors declare that they have no competing interests.

## Authors' contributions

CY carried out all the RNA expression array studies, collated all data and performed the biostatistical and informatic analyses, interpreted all the results and generated all the figures and tables pertaining to the expression array studies, and produced a preliminary draft of the manuscript. VF obtained and processed frozen primary tumors, generated all DNA extracts and many of the RNA extracts, carried out all the array CGH studies and participated in the statistical and bioinformatic analyses of these results, and contributed to drafting the manuscript. RR, JF, AH and DHM designed, supervised and/or conducted all of the biostatistical, clinical and informatic analyses supporting this study. JWG, KC, SHD, FS, ST, and AP provided all of the breast cancer study samples and/or contributed to the RNA and DNA processing of these samples. DGA helped conceive the entire study, provided laboratory support, developed all methods for and supervised the performance of all array CGH analyses, and helped interpret all results. CCB conceived the study design, identified and secured all breast cancer study samples, coordinated all DNA and RNA studies and their analyses, formulated all conclusions and drafted the final manuscript.

## Supplementary Material

Additional file 1 A .pdf file containing a figure showing a comparison between the two age cohorts in array CGH parameters: number of break points, number of chromosomes with break points, number of amplifications, number of chromosomes with amplifications, whole chromosome changes, the fraction of genome gained, the fraction of genome lost and the fraction of genome altered. Click here for file

Additional file 2 An Excel file containing a table presenting the gene sets used for GSEA. All gene sets were mapped to corresponding UniGene symbols for input into the GSEA software. Click here for file

Additional file 3 An Excel file containing a table presenting the PAM-derived age signature selected on the bases of minimizing individual cross-validation errors for both old and young cohorts. The 'o score' and the 'y score' represents a probe's contribution to the classification into the corresponding age cohorts. Click here for file

Additional file 4 An Excel file containing a table presenting the PAM-derived PR signature selected on the bases of minimizing individual cross-validation errors for both PR-negative and PR-positive groups. The 'neg score' and the 'pos score' for each probe denotes its contribution to the classification of the PR status of a particular tumor. Click here for file
