# Supplementary material for: Aging impacts transcriptomes but not genomes of hormone-dependent breast cancers
Source: Breast Cancer Res. 2007 Sep 12;9(5):R59. doi: 10.1186/bcr1765 (PMC2216076; doi:10.1186/bcr1765)
Supplement: Additional file 1 — A .pdf file containing a figure showing a comparison between the two age cohorts in array CGH parameters: number of break points, number of chromosomes with break points, number of amplifications, number of chromosomes with amplifications, whole chromosome changes, the fraction of genome gained, the fraction of genome lost and the fraction of genome altered. [file bcr1765-S1.PDF]

Number of Break Points 0.430537

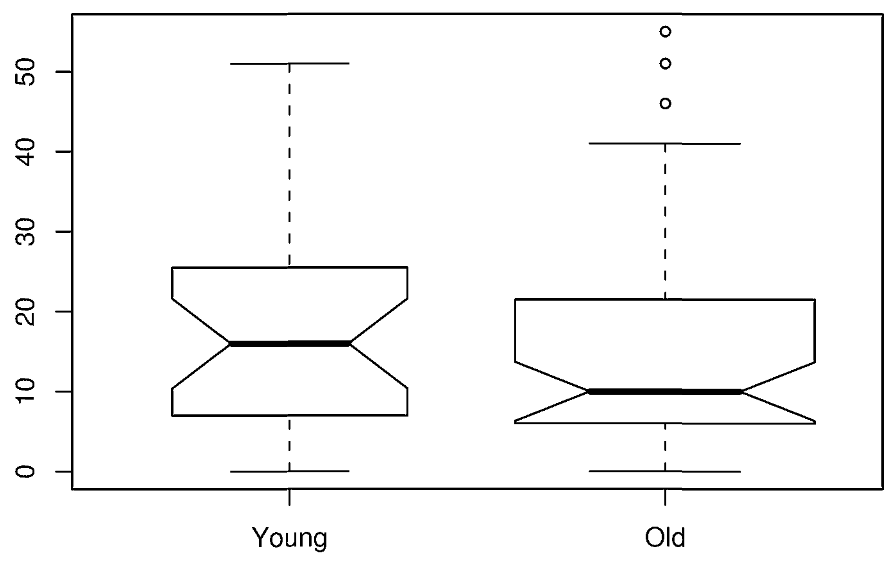

Number of Chrom with Break Points 0.326412

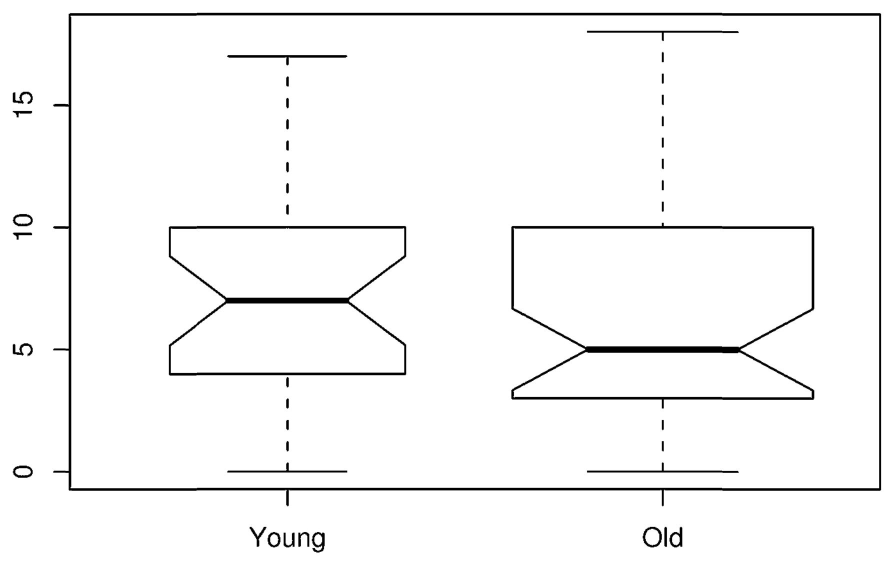

Number of Amplifications 0.176726

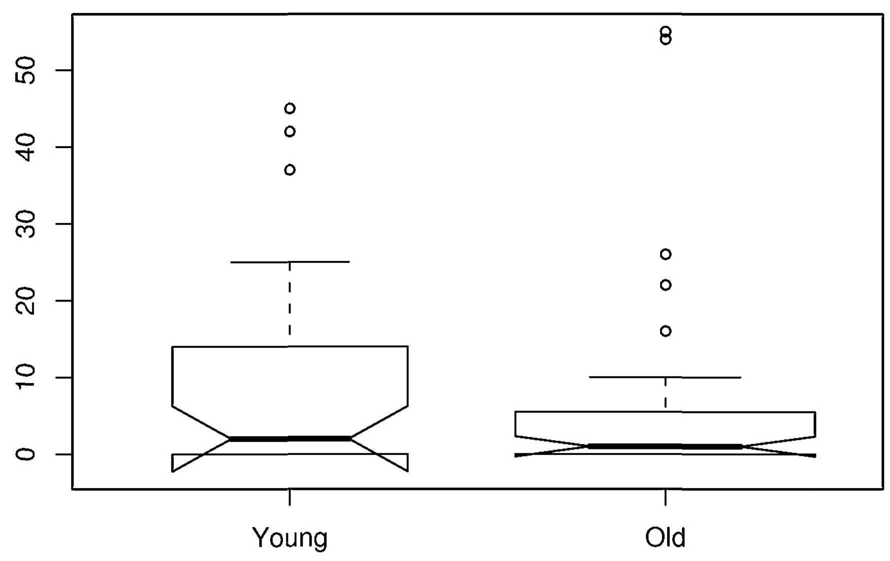

Number of Chrom with Amplifications 0.135504

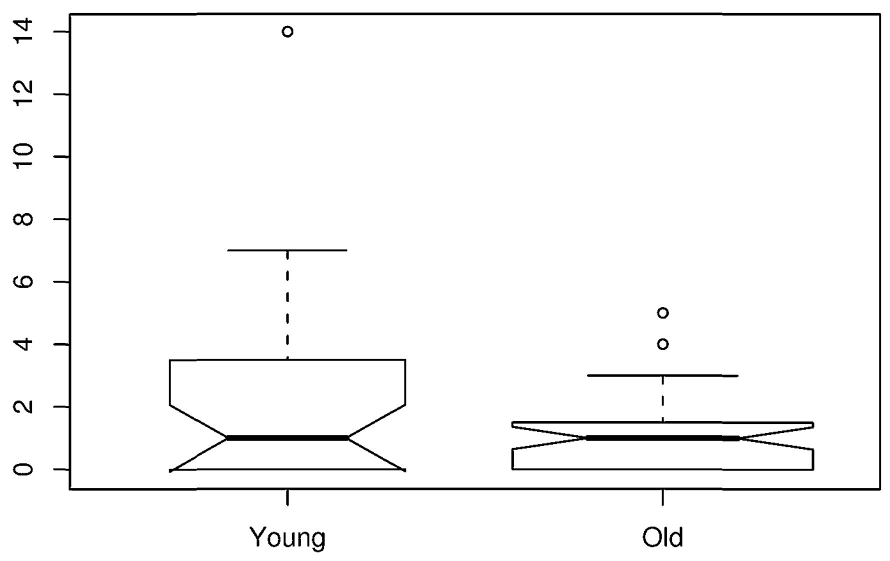

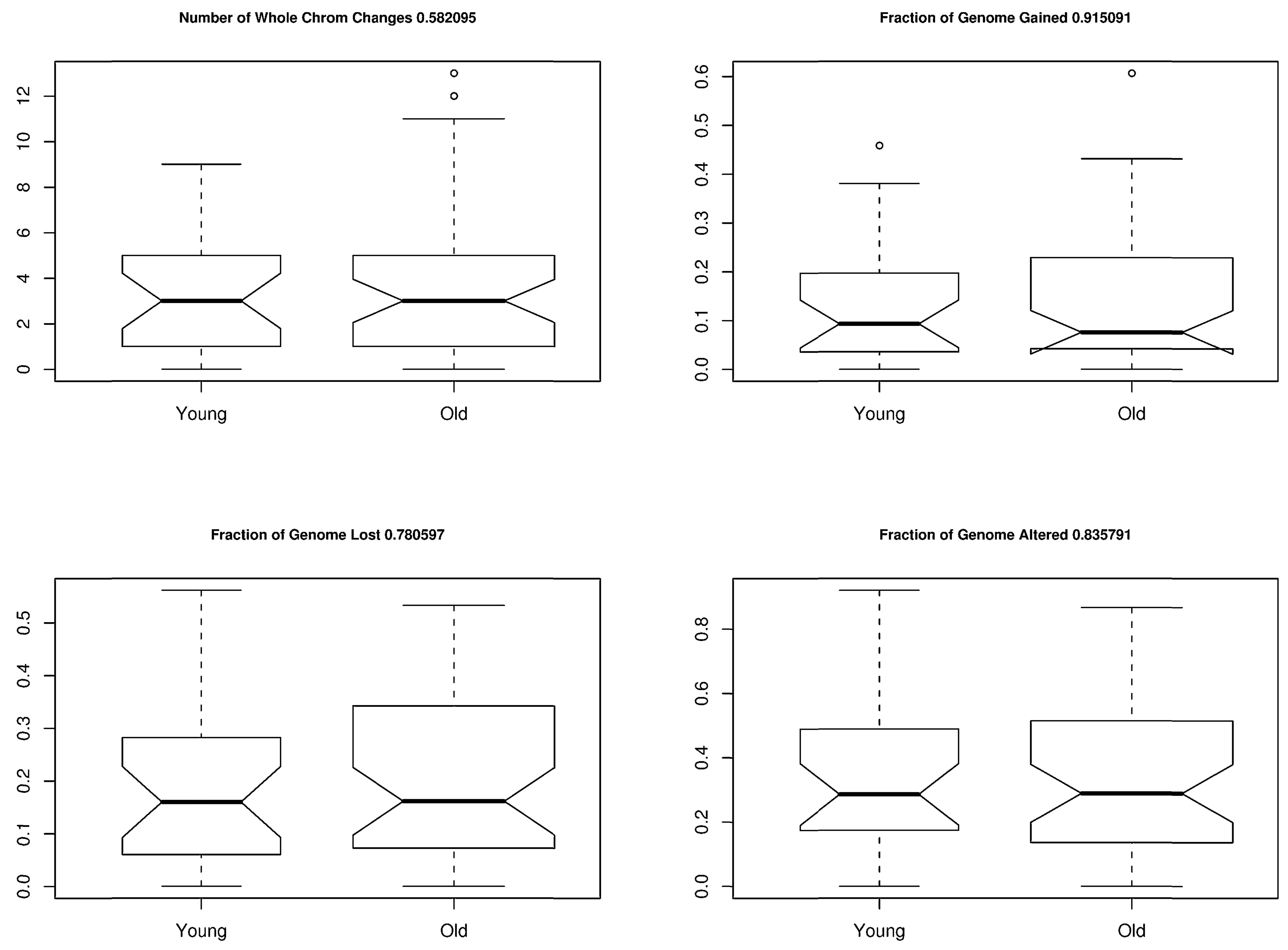
Number of Whole Chrom Changes 0.582095
0
2
4
6
8
10
12
Young
Old
Fraction of Genome Gained 0.915091
0.0
0.1
0.2
0.3
0.4
0.5
0.6
Young
Old
Fraction of Genome Lost 0.780597
0.0
0.1
0.2
0.3
0.4
0.5
Young
Old
Fraction of Genome Altered 0.835791
0.0
0.2
0.4
0.6
0.8
Young
Old
